# miRNAs as Predictors of Barrier Integrity

**DOI:** 10.3390/bios13040422

**Published:** 2023-03-26

**Authors:** Judit Bovari-Biri, Kitti Garai, Krisztina Banfai, Veronika Csongei, Judit E. Pongracz

**Affiliations:** 1 Department of Pharmaceutical Biotechnology, Faculty of Pharmacy, University of Pecs, 2 Rokus Str, H-7624 Pecs, Hungary; 2 Szentagothai Research Centre, University of Pecs, 20 Ifjusag Str, H-7624 Pecs, Hungary

**Keywords:** miRNA, exosome, barrier

## Abstract

The human body has several barriers that protect its integrity and shield it from mechanical, chemical, and microbial harm. The various barriers include the skin, intestinal and respiratory epithelia, blood–brain barrier (BBB), and immune system. In the present review, the focus is on the physical barriers that are formed by cell layers. The barrier function is influenced by the molecular microenvironment of the cells forming the barriers. The integrity of the barrier cell layers is maintained by the intricate balance of protein expression that is partly regulated by microRNAs (miRNAs) both in the intracellular space and the extracellular microenvironment. The detection of changes in miRNA patterns has become a major focus of diagnostic, prognostic, and disease progression, as well as therapy-response, markers using a great variety of detection systems in recent years. In the present review, we highlight the importance of liquid biopsies in assessing barrier integrity and challenges in differential miRNA detection.

## 1. Introduction

**Figure 1:**
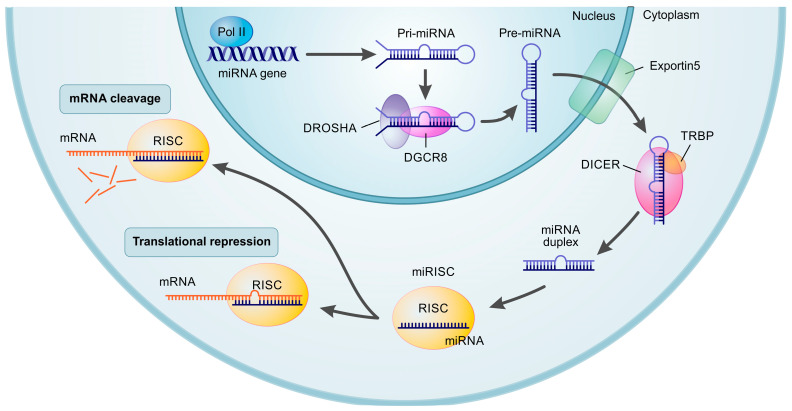
miRNA biogenesis and function.

**Figure 2:**
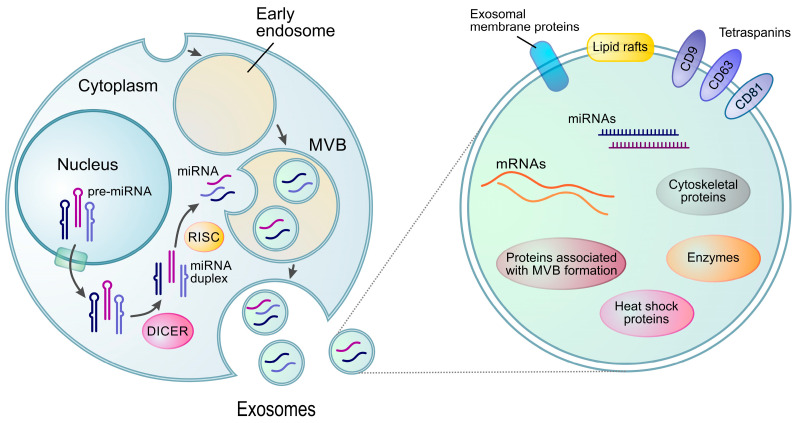
Extracellular vesicles—the production and contents of exosomes.

The early detection of illnesses, precise clinical diagnosis, therapy selection, observation of treatment responses, or prediction of adverse reactions to therapy requires both an understanding of the molecular and biological characteristics of the disease and appropriate molecular markers associated with disease progression and drug response. Traditionally, clinical pathology focuses on the molecular analysis of tissue biopsies that often require invasive sampling procedures with little chance for continuous monitoring. Moreover, tissue biopsies are often unsuitable in many cases of disease and treatment monitoring. In contrast, the use of liquid biopsies allows for frequent sampling of body fluids, including blood serum, plasma, and urine, and allows for a search for molecular markers associated with characteristic tissue malfunctions and therefore the identification of specific diseases. A molecular family that is suitable for monitoring diseases in solid tissues and body fluids alike is miRNAs. The large family of miRNAs represents short, single-stranded, non-coding RNAs with a length of about 19–25 nucleotides. Based on current estimates, the human genome encodes approximately 2300 miRNAs (miRbase V22) [1]. Different sets of miRNAs modulate gene expression at the post-transcriptional level in different cell types and tissues [2,3] (Figure 1). In contrast to freely circulating miRNAs, miRNAs in extracellular vesicles (EVs) (Figure 2) are well protected from RNAses, environmental changes, including extreme pH, and consequently from degradation. Therefore, EV-derived miRNAs can become reliable molecular markers in clinical applications. These EV-packaged miRNAs play a crucial mediatory role in intercellular communication, being secreted by and transferred to varied target cells [4]. Due to the inter-environmental communication, the targeted cells reciprocally regulate the EV-producing cells. The maintenance of a reliable balance is crucial to keep the various barriers intact. An imbalance in miRNA production results in damaged barrier functions leading to tissue dysfunction and disease. Not surprisingly, miRNAs have been identified as promising candidates for biomarkers, as well as therapeutic targets, in a great variety of diseases [5,6,7]. The challenge of miRNA detection and determination has become the focus of miRNA research.

In the present review we focus on EV-derived miRNAs associated with diseases resulting from modified barrier functions. We shall also summarize recent advances in methodologies suitable for the detection of miRNAs as biosensors.

## 2. miRNAs, EVs, and Physical Barriers

### 2.1. miRNA Biogenesis and Functions

miRNAs are produced by defined biogenesis pathways [8,9,10,11] and then, the transcripts are processed to finally form the miRNA-associated RNA-induced silencing complex or RISC [12,13]. RISC targets mRNA using a complementary, “imperfect” base pairing mechanism. The nucleotides 2–8 at the 5′-end of the miRNA serve as the targeting sequence (TS) and bind to the target site in the 3′-untranslated region (UTR) of target mRNAs [14]. Silencing the target gene can be achieved via either translational inhibition or mRNA degradation [15,16]. Alternatively, miRNAs, by silencing one specific pathway, can up-regulate the transcription of certain target mRNAs that control the target genes [17]. According to a recent estimate, 60% of all protein-encoding genes are regulated by miRNAs [18]. Intricate studies of miRNA functions have revealed that a single miRNA can potentially target numerous mRNAs, whereas one mRNA can be targeted by multiple miRNAs, reflecting the complexity of gene expression regulation [19]. An analysis of diverse biological pathways in a great variety of tissues has further emphasized the complexity of gene expression and its regulation by the miRNA system. As miRNAs are evolutionarily conserved [20,21], it is not surprising that deregulation of the miRNA system affects a great variety of physiological and consequently pathological processes [11,22].

### 2.2. miRNAs in Extracellular Vesicles (Exo-miRNAs)

While miRNAs are found within the intracellular and the extracellular space, including in the blood plasma [23], serum [24], urine [25], breast milk [26], and even in feces [27], they have also been detected in the concentrated and protected microenvironment of EVs, along with many other molecules, such as proteins and DNA. Based on the origin and size of EVs, three main groups have been identified (Table 1) [28]. All EVs are formed through budding from the plasma membrane and specific membrane markers identify the separate species of EVs (Table 1).

Protected by a phospholipid bilayer within EVs [28], exo-miRNAs are able to travel in the blood stream or other bodily fluids and target a great variety of cell types to modify their function. The surface markers in the EV membrane aid their binding to target cells. Depending on the subtype of EVs, they can be enriched with Endosomal Sorting Complexes Required for Transport or ESCRT components (e.g., vacuolar sorting proteins (VPSs) VPS24, VPS32, and VPS36) [29], the molecular scaffolds of tetraspanins (e.g., CD81, TSPAN9, and TSPAN14) [30], annexins (ANXA7) [31], flotillins [32], and integrins (ITGA3) [33]. Functional studies have raised the possibility that exosomes and microvesicles function via different biological pathways. While exosomes are enriched in signaling proteins (e.g., proteins implicated in ESCRT, syndecan signaling, and membrane trafficking), microvesicles are enriched in enzymes and proteins regulating gene expression and translation [34].

### 2.3. Maintenance of Physical Barriers

**Figure 3:**
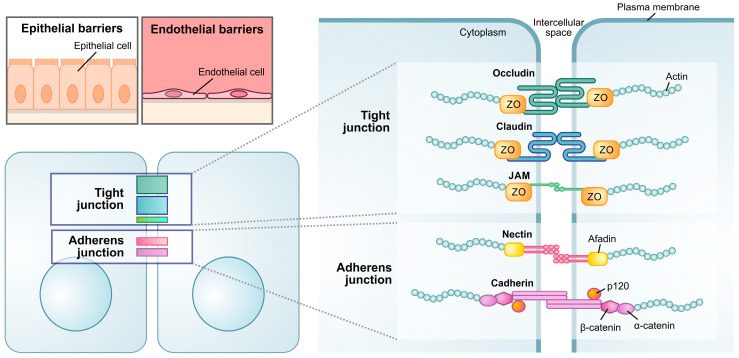
Structures of epithelial and endothelial barriers.

The epithelial cells form layers to act as selective gateways between the outside environment and underlying tissue [35], while endothelial cells are one of the most important components of blood vessels [36] (Figure 3). Endothelial junction proteins maintain tissue integrity, but also allow for vascular permeability, leukocyte extravasation, and angiogenesis. The Apical Junctional Complex (AJC) consists of tight (TJ) and adherens junction (AJ) protein complexes [37,38]. The AJC ensures tight connections of polarized epithelial and endothelial cells in the intestinal mucosa and in the walls of blood vessels, respectively. The AJC maintains structure, homeostasis, and the function of the barrier [39]. Tight junctions are multi-protein complexes localized at the apical side of lateral cell membranes. Currently, there are more than 50 TJ-associated proteins that regulate selective membrane permeability for molecules based on charge and size [40].

Occludin, claudins, and junctional adhesion molecules (JAMs) constitute the main protein complexes of TJs [41]. The adaptor proteins zonula occludens (ZO-1, ZO-2, and ZO-3) are linked to the cytosolic C-terminus of the trans-membrane proteins [42]. The adaptor proteins are also anchored to the actin cytoskeleton [43,44].

The important AJ transmembrane proteins belong to the cadherin family [45]. The cytoplasmic tail of E-cadherin is linked to the actin cytoskeleton, and other signaling elements through many peripheral membrane proteins. The junctional complexes are summarized in Table 2.

### 2.4. Maintenance of Tight Junctions

The function of tight junctions is challenged by a variety of factors, including toxins, pathogens, or inflammation and cancer, which can lead to a loss of barrier integrity and finally to tissue damage [46,47,48,49]. Both the loss and assembly of tight junctions are regulated by numerous signaling pathways that involve a great variety of molecules, including protein kinases, phosphatases, G-proteins, and the regulators of their expression at both mRNA and protein levels [50,51]. The post-transcriptional regulation of tight junction proteins also involves miRNAs, which can directly affect the level of transmembrane proteins [52,53]. The regulation of occludin by miRNA was examined in intestinal epithelial tight junction permeability. Using bioinformatics algorithms, miRNA-binding sites on the 3′UTR of occludin mRNA [54] predicted three potential miRNA-binding motifs on the occludin 3′UTR for miR-122a, miR-200b, and miR-200c [55]. The same methods indicate that the miRNA regulation of molecules in TJs and AJs is also possible. Using in silico analysis and then sometimes confirmational laboratory experiments, several miRNAs have been identified in the regulation of specific diseases, and some of them were also associated with barrier functions (Table 3). Table 4 summarizes miRNAs that directly or indirectly regulate the translation of specific barrier proteins.

### 2.5. Defective Intestinal Epithelial Barrier

The epithelial tight junction (TJ) barrier is defective in Crohn’s disease, inflammatory bowel disease, ulcerative colitis, infectious diarrheal syndromes, and celiac disease leading to increased intestinal permeability, which contributes to intestinal inflammation. The main barriers in these cases are the epithelial cell layers of the gut and the intestinal immune system. miRNAs involved in this regulation are summarized in Table 3, while miRNAs targeting barrier proteins are summarized in Table 4. The specific role of miRNAs in intestinal barrier integrity is complex. One of the best studied miRNAs in intestinal barrier integrity is miR-21, which was initially demonstrated to target the TJ proteins occludin, claudin-2, and ZO-1, leading to their reduced expression and subsequently impaired barrier integrity [96]. In contrast, miR-21 has recently been shown to increase the expression of occludin via a ROCK1-dependent mechanism [97], suggesting a positive role in the TJ barrier function and reduced inflammatory response.

### 2.6. Defective Lung Epithelial Barrier

A defective barrier function has been identified in many lung diseases, including asthma [98], chronic obstructive pulmonary disease, and acute pulmonary disease, which can be the direct consequence of viral or bacterial infections [98]. The modulatory miRNAs involved in the process are summarized in Table 3, while miRNAs targeting barrier proteins are summarized in Table 4. In acute inflammatory pulmonary injury, miR-27a-5p and miR-27a-3p were implicated, while in other studies, miR-27a was associated with VE-cadherin regulation. Reduced VE-cadherin levels were shown to lead to vascular leaks and delayed recovery from ischemic injury [99]. In addition, miR-27a has also been associated with blood–brain barrier (BBB) integrity [100].

### 2.7. Defective Urinary Bladder Epithelial Barrier

The urothelium plays a central role as a barrier between urine and the underlying bladder. Disruption of the urothelial barrier initiates a cascade of events in the bladder that can lead to interstitial cystitis (IC). Biomarker candidate miRNAs listed in Table 3, miR-373-5p and miR-6766-5p [78], were described as potential regulators of disease development. While the current literature associates miR-373-5p with the regulation of ZO-1 levels [101], miR-6766-5p regulates alpha-smooth-muscle actin indirectly via the TGFβRII–SMADS (transforming growth factor β receptor II–small mothers against decapentaplegic protein) pathway [102].

### 2.8. Defective Blood–Brain Barrier

Perhaps one of the best studied barriers is the BBB. Diseases, including epilepsy, multiple sclerosis, Alzheimer’s Disease, autism spectrum disorder, and stroke, are associated with impairment of the endothelial barrier function, contributing to disease development and progression. Disease-associated miRNAs are summarized in Table 3. In the above diseases, the endothelial barrier is breached contributing to disease development and progression. Several miRNAs have been identified in the regulation of barrier integrity, directly affecting TJ and AJ protein expression (Table 4). miR-27a, miR-210, and miR-373 are all involved in various brain diseases by modifying VE-cadherin, occludin, and ZO-1 respectively, just to specify a few.

Due to complex and sometimes contrasting evidence presented in the literature for the role of miRNAs in the regulation of disease-associated barrier proteins, specific and sensitive detection methods had to be developed.

## 3. Biosensors and miRNA Detection

The importance of miRNA [103] biomarkers in diagnosis, prognosis, and treatment responses of various diseases has gained momentum in the past couple of years. Two major areas need to be considered during the search and detection of biomarkers: one is the selection of the target miRNA molecules, while the other is the detection method itself. Extensive efforts have been made to detect miRNAs in liquid biopsies reliably, specifically and at a low cost [104], to make such methods are suitable for clinical application. Table 5 contains the main combinations of assays and biological recognition elements.

**Figure 4:**
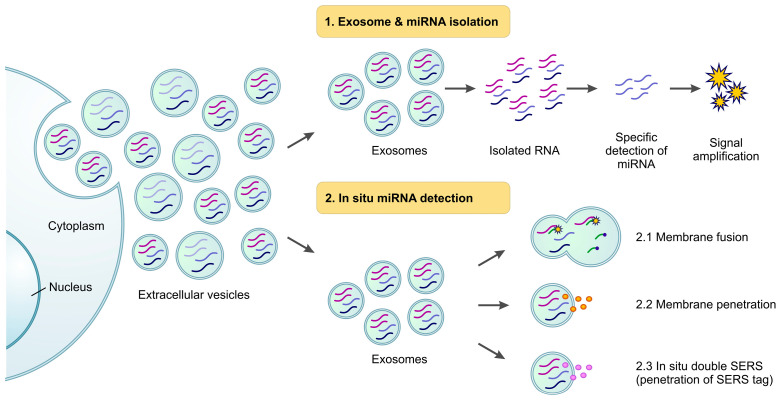
Detection of exo-miRNAs in clinical samples.

Categorizing detection methods is complex. The main criteria include whether the target miRNA is isolated from the sample or the target is detected in situ within the exosome (Figure 4). Additional challenges include the amplification of the target or magnification of the generated signal.

### 3.1. Isolation of Exosomes and Exo-miRNAs

The first step is the isolation of exosomes, followed by the specific detection of exo-miRNAs.

There is a range of techniques to isolate EVs and their subtypes, including exosomes [118]. Although the current gold standard for EV isolation is ultracentrifugation [119], it is not suitable for a clinical diagnostic lab-on-a-chip protocol. Size exclusion and affinity chromatography separate EVs from soluble proteins in liquid biopsy samples and can deliver highly purified EVs in an efficient and standardized manner. Additionally, they can fit the required sizes even for a microfluidic biochip, allowing for rapid separation over a wide range of biofluids and sample volumes.

The detection of specific exo-miRNAs in exosomes as diagnostic markers from a few microliters of liquid biological samples is challenging. Technically, there are two options (Figure 4): to isolate exosomes followed by RNA purification and the specific detection of miRNAs (3.1.1) or to measure miRNA levels in intact exosomes (3.2.1).

As the amount of the target exo-miRNAs is extremely small, the next question in both approaches is how to detect specific molecules below the nano-molar (nM) scale. Once the target molecule is captured, a signal needs to be emitted, and the generated signal needs to be sufficiently amplified to become detectable.

Nucleic acids, including miRNAs, have a specific sequence; therefore, the solution for specificity is relatively simple—a complementary sequence needs to be generated for the target. As simple complementarity-based sequence binding is not going to generate a signal, a measurable molecular alteration is required. Such molecular alterations can be a hairpin structure. Once the target molecule binds to the complementary sequence, a signal, either fluorescent, electrochemical, or other, is emitted due to changes in the molecular structure (hairpin opens). Finally, the emitted signal needs to be amplified to be detected. In the analysis of the contents of exosomes, Raman spectroscopy has also become popular as each molecule has a unique Raman spectrum; consequently, it can be detected without specific targeting and is “label free” at the single-particle level [120,121]. A source of high-intensity light is applied to the sample, and incident photons are scattered by molecules. The frequency and intensity of scattered radiation reveal the quality and quantity of the sample, respectively [122].

Depending on the combination of the above techniques and methods, the sensitivity of detection can be as low as the pico-(p) (10^−12^), femto-(f) (10^−15^), atto- (a) (10^−18^) and zepto-(z) (10^−21^) molar level.

#### 3.1.1. Direct Detection of Isolated Exo-miRNAs [123,124] and Sequencing [95]

##### Direct Amplification of Exo-miRNAs

From an isolated EV pool and from the separated exosomes and their purified RNAs, miRNAs can be detected via quantitative reverse transcription dependent real-time polymerase chain reaction (qRT-PCR), digital PCR, and next generation sequencing (NGS), which are accurate methods of miRNA detection. Unfortunately, they have several drawbacks. Some of the disadvantages include the lack of absolute quantification of previously identified and disease-specific miRNAs. The above methods can also provide false positive results, and they rely on labor intensive methodologies, as well as require expensive equipment, which makes them less suitable for clinical application.

##### Amplification-Free Direct miRNA Detection

Amplification-free methods have also been developed for miRNA detection [125]. Amplification-free methods use unique fluorescent barcodes that enable the direct, digital detection of hundreds of different target miRNAs in a single run. Unfortunately, the equipment is expensive, and data analysis requires extensive statistical evaluation. Custom-made chips can be designed, once the clinically relevant miRNA sets are identified, but can still be costly for routine detection in clinical samples.

#### 3.1.2. Exo-miRNA Detection Using Novel Signal Amplification Strategies

Although initial detection methods have already made use of the nucleic acid nature of miRNAs, there was and still is demand for novel detection methods using the same nucleic acid-based strategy but with added signal amplification for more sensitive detection.

##### Nucleic Acid-Based Strategies

Nucleic acid-based strategies include nucleic acid-based capture probes of a great variety, including individual and assembled, natural, or synthetic probes.

Nucleic acid capture probes

Natural individual probes [126,127,128,129,130,131] are designed to complement the target sequence. The complement sequences to specific miRNAs are strategically positioned into a loop structure, where the loop ends complement each other and form a hairpin structure. One end is labeled with a fluorescent dye, while the other is labeled with a quencher. In the presence of the target sequence, the hairpin structure opens the loop structure, and fluorescence can be detected. If there is no target sequence in the sample, the natural hairpin structure remains in place and the quencher absorbs fluorescence. If the signal is too weak, then enzymatic signal amplification methods can be performed [132] increasing the detection limit.

Synthetic individual probes [133,134] are designed to improve thermostability and selectivity. Synthetic probes are made with sugar or polypeptide backbones attached to nucleic acid sequences complementary to the target miRNA. The probes with the sugar backbone are made with Cy3-labeled capture probes on gold nanopillars that increase sensitivity to the aM range [135]. The advantage of peptide backbone probes is their lack of electrostatic repulsion to nucleic acids during base-pairing interactions, which increases their sensitivity.

Based on the natural characteristics and assembly processes of nucleic acids, DNA can be designed to form nanostructures. Such assembled capture probes are nanostructures, which can be programmed predictively using various reporter groups, including electrochemical sensing and fluorescence, etc. The sensitivity of such capture probes varies between aM and pM [136,137,138].

DNA self-assembly induced signal amplification

Normally, no enzyme is required for signal amplification reactions of DNA self-assembly. Hybridization chain reaction (HCR) [139,140,141], catalytic hairpin assembly (CHA) [142,143], and the toehold-mediated strand displacement reaction [144] are examples of the DNA self-assembly methods. In HCR isothermal signal amplification, where two nicked hairpin DNAs and a single-stranded initiator DNA generate and alter the resulting hairpins, the signal can be further amplified by using gold electrodes and electrochemical signal reporting to increase sensitivity to the aM range. CHA consists of two DNA strands that can form a hairpin structure. In the presence of the trigger strand, one DNA strand opens, and the opened strand can open the other strand, which then forms the thermodynamically stable double-strand. Sensitivity and specificity of the CHA method can be improved by a step polymerization hairpin assembly system and with biotin and the biotinylated tandem product to generate sensitive electrochemical signals in the fM detection range [145]. The various signal amplification methods can increase the detection limit between aM and fM [146,147].

Isothermal amplification

In contrast to the signal amplification method based on DNA assembly, this is an enzyme-assisted method that can trigger the amplification reaction of DNA at a constant temperature with the assistance of different isothermal amplification enzymes, thus achieving exponential signal amplification. Both the rolling circle amplification (RCA) [148,149,150,151] and the exponential amplification reaction (EXPAR) [152] are examples of the isothermal signal amplification methods and have a detection limit mostly in the fM range.

##### Nucleic Acid-Synergized Strategies

Nanomaterials

Dimensions of nanomaterials range between 1 and 100 nm. The material itself can be metallic, inorganic, and organic. The advantage of using nanomaterials includes flexible and modular structures, a large surface area, small size, and good biocompatibility [153]. In addition, some materials have shown properties suitable for biological sensing, such as enzyme-mimicking features, fluorescent properties, and superparamagnetic behavior. In the detection of exo-miRNAs, gold nanoparticles (AuNPs), carbon dots (CD) [154,155], carbon nanotubes (CNTs) [156], and metal-organic frameworks (MOF) [157] are widely used. The amplified fluorescence, luminescence, or electrochemical signals allow for the simultaneous analysis of more than one exo-miRNA with detection ranging from aM and fM to nM.

Microfluidics

As clinical demand increases to detect exo-miRNAs from liquid biopsies, microfluidic devices, including chips and paper strips, have become increasingly popular allowing for sample processing and analysis on the same microfluidic platform [158]. Ideally, “chip” platforms are integrated systems, where exosomes are isolated from the fluidic sample using anti-CD63-coated magnetic beads [123]; then, miRNAs are isolated, amplified, and detected [159].

Paper-based test strips use a lateral flow assay (LFA) assisted by DNA barcode sequences, where the PCR product is captured by the immobilized DNA probes on LFA and detected using streptavidin-coated gold nanoparticles [160].

##### Signal Sensing Strategies

While in several systems, the detection of miRNAs relies on fluorescent sensors, the detection of exosome-derived miRNAs is less reliable using fluorescence. Primarily, this is because the fluorescence signal can be reduced due to self-quenching and fluorescence background signal interference reducing both sensitivity and the detection limit.

SERS (Surface-Enhanced Raman Scattering)

SERS is a surface-sensitive technology of scattering enhanced by nanostructures [161]. The original SERS method had to be modified for biological samples using gold and/or silver nanoparticles. The SERS technique is highly sensitive and has a low background-to-noise ratio making it suitable for exo-miRNA detection in clinical samples. As the normally used SERS signal tags in a biological environment cause instability on the surface, novel analysis strategies were developed where SERS reporter elements and duplex-specific nuclease-assisted signal amplification can be combined [162].

All the SERS reporter elements (signal reporter, recognition, and separation element) have been modified for increased sensitivity and specificity. For example, Rhodamine 6G was attached to gold nanoparticles encapsulated in silver and gold (AgAu) alloy nanoparticles in the signal reporter, while the separation element is combined with streptavidin-coated microbeads using a target miRNA complementary DNA sequence as a capture probe. To amplify the generated signal, duplex-specific nuclease-assisted signal amplification is used to make exo-miRNA detection possible within the fM range. To increase the detection sensitivity of extremely low-concentration biomolecules, 3D plasmonic nanostructures are used with a significantly amplified SERS effect. This method allows for identification of the Raman spectral fingerprint of a single molecule [163].

SPR (Surface Plasmon Resonance)

Surface plasmon resonance (SPR) is an optical sensing technology [164]. It is highly sensitive to the refractive index of the surface media. In SPR biosensing technology, SPR is combined with a biological immunoassay, significantly improving sensitivity [165]. Due to its labor-intensive sample preparation and expensive instrumentation, its clinical application is limited.

Electrochemical detection

Electrochemical-based methods have recently attracted significant interest, due to their high sensitivity and specificity, short reaction time, simple operation procedures, and wide dynamic range. The great variety of electrochemical biosensors provide effective, fast, economical, and amplification-free miRNA detection.

Several variations of electrochemical detection methods have been developed, including superparamagnetic gold-loaded nanoporous iron oxide nanomaterials in electrochemical sensors or catalyzed hairpin assembly, which was subjected to streptavidin–biotin interactions [166], greatly improving both the specificity and sensitivity of exo-miRNA detection. This method was able to reduce the detection limit without enzymatic amplification [167]. Additionally, a two-step competitive hybridization assay was developed combined with gold nanoparticles [168] for the ultrasensitive detection of exo-miRNAs in complex clinical samples.

### 3.2. In Situ Detection

In most detection strategies of exo-miRNAs, the first step includes exosome isolation from the mixed EV pool of the biological sample. Exosome isolation is followed by RNA extraction that may result in the loss or contamination of miRNAs, making an in situ approach more desirable.

#### 3.2.1. Membrane Fusion

The most used in situ detection method involves the membrane fusion of exosomes. This way, the detection of exo-miRNAs can be performed without disrupting the membrane of the exosome, protecting the integrity of nucleic acids and making the reaction confined by the fused membranes, which increases the local concentration of the signal molecule and the sensitivity of the reaction and reduces the limit of detection [169,170,171].

#### 3.2.2. Penetration

The penetration method frequently uses gold nanoparticles and defined DNA nanostructures to penetrate exosomes for the specific detection of miRNAs [172,173].

#### 3.2.3. In Situ Double SERS (Surface-Enhanced Raman Scattering)

The double SERS biosensors accurately and quickly separate exosomes from serum samples and directly detect specific miRNAs in exosomes in situ where the SERS tag can be transferred to exosomes [174,175]. The methods that were generated to provide electromagnetic hotspot-enhanced SERS signals make exo-miRNA detection simple and highly sensitive, making this method suitable for clinical application.

## 4. Discussion

Diagnostic and prognostic markers must be reliable to provide the basis for clinical decision-making. To be able to reach the clinical application stage, the identification of clinically relevant miRNAs requires careful planning and extensive laboratory research. Cells of healthy and diseased tissues contain different types of miRNAs, and they also release a different set of miRNAs as the content of exosomes [176]. The planning of clinical sample collection, the method of sample taking, and finally, the storage, especially long-term storage of the collected samples, are extremely important to identify a clinically relevant biomarker. As the accuracy of exosome isolation and purification and then RNA isolation and miRNA profiling all depend on the techniques, the qualitative identification of exo-miRNAs as disease-associated markers is not only the first stage but is also the decisive stage, often in need of large sample cohorts [177].

In the next research stage, the clinical evaluation of potential diagnostic markers requires accurate enumeration techniques, and the methods need to be reliable and sensitive to be easily used in a clinical laboratory setting. The recently developed in vitro miRNA detection methods have been classified into three major groups, including those that detect biological recognition elements, use added micro-/nanomaterials, or use signal transduction/readout elements. Grouping biosensors can also focus on the actual detection methodology, including hybridization and amplification [178], colorimetric [108], fluorescent [109], optical [114,115], electrochemical [105,106], and magnetic nano-techniques [104].

The overall time of detection and the required amount of sample are also important. While the fastest (1 min detection time) needs a large amount of sample with relatively high miRNA content [179], a low sample amount needs nearly a day to detect the target miRNA to achieve acceptable sensitivity (10^−17^) [180]. Generally, most techniques need a couple of hours to complete miRNA-specific detection, and they are mostly not multiplex methods making exo-miRNA-based differential diagnosis difficult. The difficulty of finding disease-specific appropriate biomarkers has long been recognized. The entry of miRNAs into the clinical diagnostic and prognostic landscape, as well as the therapeutic target scene, highlights further the complexity of disease-associated gene expression regulation. Although the use of miRNAs in clinical applications has been quickly recognized, the complex role and small amounts of miRNAs in the regulation of health and diseases have made it difficult to reliably apply their detection in diagnosis.

It has come to light that those miRNAs, which are important in disease regulation, can also be found and safely preserved in EVs. As EVs can be isolated from body fluids, the role of EVs in diagnostics has increased in recent years. EVs isolated from human blood sera or plasma, amongst other molecules, contain miRNAs. The smallest of EVs are the exosomes, and their miRNA content is currently the most thoroughly investigated, where exo-miRNAs are safely preserved for clinical analysis. Exo-miRNAs carry a unique load of information about the patient’s internal microenvironment and its regulation, providing an intricate view of the patient’s health status once the biomarkers can be reliably associated with specific diseases and detected and quantified dependably. Consequently, the selected methodology is just as vital as the identification of the target exo-miRNA itself. Moreover, it is often a group of exo-miRNAs, rather than a single molecule, which describe the disease or the disease stage accurately. As miRNAs and exo-miRNAs are associated with specific diseases, it is becoming clear, that the same miRNAs are associated with a variety of diseases, and the targets of these miRNAs are similar or even the same barrier proteins but in different tissues.

Not surprisingly, extensive efforts have been made to determine and select the right miRNAs as biomarkers and to find the right probes and technology that identifies the biomarkers in a highly sensitive assay system. Such in vitro detection methods require high sequence homology, high accuracy, multiplexity, high sensitivity, and low cost to be used in clinical settings.

Due to the diversity of methods, the question is rather which miRNA can be reliably associated with barrier integrity. If miRNAs, especially exo-miRNAs safely wrapped into EVs of body fluids, can be used to predict barrier dysfunction caused by diseases or drugs, this could greatly contribute to the prevention of disease progression and safe selection of medications.

If the currently available techniques are to be used in the routine clinical evaluation of patients’ health status, the simplest, fastest, and most accurate method is the label-free Raman technology, using double SERS biosensors. This method is suitable for use with biological samples and can directly detect specific miRNAs in exosomes in situ. While this technique sounds ideal, Raman spectra of all clinically relevant exo-miRNAs have to be pre-determined and stored in databases.

## 5. Conclusions and Future Perspectives

While the recent literature is bursting with disease-associated miRNAs, confirmatory clinical studies are few and far between. This is particularly true of exo-miRNAs that require the first and ambiguous step of EV isolation preceding the detection of miRNAs. Often the detection methods are either not sensitive enough or, even more importantly, have not reached the simplicity and reliability required in clinical laboratories. Until then, more research is needed to incorporate exo-miRNAs as disease markers related to barrier functions into the already existing and proven miRNA-identifying detection methodologies. The selection of the specific method depends on several parameters, the amount of the miRNA to be detected, the biological type of the sample, where proteins and other biomolecules can interfere with detection sensitivity, and the number of miRNAs that regulate barrier function in parallel, and consequently, detection requires multiplex assays. Based on our current understanding of disease-specific miRNA markers, a single miRNA is highly unlikely to provide a definitive diagnosis. Most likely, a group of well characterized exo-miRNAs will be required to describe the status of disease-associated barriers. The advantage of nanomaterials comes naturally. The size of exosomes falls between 30 and 100 nm, and therefore, nanomaterials (1–100 nm) that can penetrate exosomes and make in situ detection possible have an advantage. Nanomaterials can be applied in miniaturized biosensors, thus promising small, low-cost, portable, and user-friendly medical diagnostic point-of-care or bedside diagnostic tools. In addition, the high-surface area properties of nanomaterials allow them to be used directly as signal capture-carriers for the application of isolating and capturing disease biomarkers in the circulatory system. The ability of nanostructured materials to directly engage with the sensing environment (electrolytes, markers, etc.) accelerates target-to-detector signal transduction, which in turn improves the robustness and sensitivity of the analysis and reduces detection limits. By modifying nanomaterials, they can be applied in label-free assays for the direct detection of biological systems. Such label-free assays include Raman spectroscopy. Once the specific and individual Raman spectrum becomes known for most of the clinically relevant miRNAs, the identification of miRNAs within exosomes becomes the easiest for use in clinical diagnostics.

The future is bright for nanosensors in the exo-miRNA field, not only at the diagnostic level, but also for the careful and reliable monitoring of the response to specific drugs modifying barrier functions. Using fast, sensitive, and relatively cheap methods that are easy to use even at bedside may provide a further understanding of drug responses and may lead to the identification of markers warning medical personnel of potential side effects of the medications planned for individual patients.

## Figures and Tables

**Table 1:** Characteristics of extracellular vesicles.

Type and Size of EV	Molecular Markers	Contents
Exosome (30–100 nm)	ALIX, TSG101, CD63	Nucleic acids (DNA, mRNA, miRNA), lipids, proteins, metabolites
Ectosome (microvesicle) (100–1000 nm)	ARF6, VCAMP3	Nucleic acids (DNA, mRNA, miRNA), lipids, proteins, metabolites
Oncosome (Apoptotic body) (1–5 µm)	Cytokeratin-18	Proteins, metabolites

**Table 2:** Junctional complexes.

Junctional Complexes	Function	Main Junctional Proteins
Tight junction (TJ)	Maintain cellular polarity and establish distinct fluid compartments	Zonula occludens (ZO-1, ZO-2, and ZO-3) bound to occludin or claudins and actin
Adherens junction (AJ)	Initiation and stabilization of cell–cell adhesion, regulation of the actin cytoskeleton, intracellular signaling and transcriptional regulation	E-cadherin linked to actin cytoskeleton, VE-cadherin, catenins, and signaling elements via membrane proteins

**Table 3:** Barrier function diseases and miRNAs.

Organ	Disease	Cell Type	miRNA
Intestine	Inflammatory bowel diseases Crohn’s Disease [56]	epithelial	miR-1-3p and miR-124-3p [57]
	Ulcerative colitis [58,59]	epithelial	miR-21, miR-122a, miR-124, miR-191a, miR-212, miR-675, miR-874, miR-93, miR-200b, miR-20a, miR-30c miR-93, miR-106b, miR-122, miR-130a, miR-132, miR-2, miR-142-3p, miR-146b, miR-155, miR-192, miR-196, miR-320, miR-10a, miR-141, miR-320, miR-346, miR-665 [60]
	Celiac disease [61]	epithelial	miR-31-5p, miR-192, miR-194, miR-449a, and miR-638 [62] miR-29c and miR-224 [63]
	Infectious diarrheal syndromes [64]	epithelial	
Lung	Asthma [65,66]	epithelial	Let-7a [67], miR-let-7f, miR-181c, miR-487b, miR-34b-5p, miR-34c-5p, miR-449a, miR-449b-5p, miR-155, miR-203, miR-221, miR-3065
	Pollen allergy [68]	epithelial	[69] miR-21, miR-146a miR-155 [70], miR-151a, miR-143, miR-124, miR-223, miR-10a, miR-572, miR-1228-, miR-483, miR-1908, miR-126, miR-92a, miR-125a, miR-19a, miR-26a, miR-106a, miR-181c, -3177
	Interstitial pneumonia [71]	epithelial	miR-193a-5p, miR-502-3p, miR-200c-3p, miR-16-5p, miR-21-5p, miR-126-3p, miR-34a-5p [72] miR-375, miR-193a, miR-106b, miR-18a, miR-15a, and miR-374a [73]
	Acute respiratory distress syndrome [74] and acute lung injury	endothelial	miR-155, miR-223, miR-146a, miR-27a/b [75], miR-34a, miR-132, miR-15a, miR-21 [76]
Urinary bladder- Urothelial Barrier	Interstitial cystitis/bladder pain syndrome [77]	epithelial	miR-373-5p, miR-6766-5p [78]
Blood-brain barrier	Alzheimer’s disease (AD) [79]	endothelial	miR-15a/16-1 [80]
			miR-501-3p [81]
	Multiple sclerosis (MS) [82]	endothelial	miR-182 [83,84]
	ischemia/stroke [85]	endothelial	miR-107 [86]
	Epilepsy [87,88]	endothelial	miR-143, miR-27a-3p, miR-101, miR-132, miR-210, miR-125a-5p, miR-126-3p, miR-98, let-7g, miR-1303, miR-132, miR-27b, miR-150, miR-150, miR-182, miR-285, miR-181c, miR-30a [89]
	Autism spectrum disorder [90]	endothelial	miR-6780a-3p, miR-1225-5p, miR-2277-3p, miR-548j-5p, miR-100-5p [91], miR-1290 [92], miR-873 [93] miR-486, miR-181b [94]

**Table 4:** miRNA-regulated barrier proteins.

miRNA	Targeted Barrier Protein	References
miR-15a, b/16-1	Claudin-2, 5	[95]
miR-16-5p	Claudin-2	[96]
miR-21, -5p	Claudin-2, occludin, ZO-1	[96,97,98]
miR-27a/b, a-3p	Claudin-2, 5, 8, VE-cadherin	[99,100]
miR-34, -a	Claudin-8, ZO-1	[101]
miR-93	Claudin-3	[102]
miR-101	VE-cadherin	[103]
miR-122a	Occludin, ZO-1	[55,104]
miR-122	Claudin-2, 8, occluding	[105]
miR-125a,b, -5p	Claudin-2	[96,106]
miR-142-5p	Claudin-1	[107]
miR-142-3p	Claudin-12, ZO-1, occludin	[108]
miR-144	Claudin-1, ZO-1	[108]
miR-155	Claudin-1, 8, ZO-1, occludin	[106,109]
miR-182	Claudin-5	[110]
miR-195-5p	Claudin-2	[111]
miR-200a	ZO-1	[112]
miR-200b	Claudin-8, ZO-1, occludin	[113]
miR-200c	Claudin-8, ZO-1, occludin	[114]
miR-200	ZO-1	[115]
miR-210	Occludin	[116]
miR-212	ZO-1, occluding	[117]
miR-373-5p	ZO-1	[95]
miR-429	Claudin-8, ZO-1, occludin	[118]
miR-874	Claudin-1, occluding	[119]
miR-1290	Claudin-1, 5, ZO-1	[120,121]

**Table 5:** Various miRNA detection methods.

Assays	miRNA	Sensitivity (mol/L)	Biological Recognition Elements
Electrochemical [105,106]	Let-7a, miR-21, miR-141, miR-122, miR-196a	10^−7^–10^−18^	ssDNA probe, ssRNA probe, hairpin-shaped probe, HCR, CHA, DSNSA, (clustered regularly interspaced short palindromic repeats) CRISPR–Cas system [104,107]
Colorimetric [108]	miR-221, miR-21, miR-141 Let 7a, -7b, -7d, miR-1178, miR-1248	10^−7^–10^−18^	CHA, EXPAR, DSNSA, hairpin-shaped probe,
			CRISPR–Cas system [104,107]
Fluorescent [109]	miR-19b, miR-21, miR-92a, miR-148, miR-146a, miR-185	10^−8^–10^−17^	ssDNA probe, hairpin-shaped probe, HCR, CHA, DSNSA, EXPAR, RCA, CRISPR–Cas system [104,107]
Luminescence [110]	miR-21	10^−8^–10^−12^	CRISPR–Cas system [104,107]
Raman scattering [111,112]	miR-21a,		
	155a, 10b, 96a,125a, 34a	10^−9^–10^−15^	Hairpin-shaped probe
Gold nanorod [113]	miR-21	10^−17^–10^−17^	Hairpin-shaped probe
	miR-200b		
Optical biosensor [114,115]	miR-133a, miR-141, miR-21, miR-155	10^−1^7–10^−17^	Hairpin-shaped probe, CHA, ssDNA probe
		10^−17^	ssDNA probe
Lateral flow [116,117]	miR-21, miR-224		

## Data Availability

Not applicable.
